# Erythrocyte Oxidative Status in People with Obesity: Relation to Tissue Losses, Glucose Levels, and Weight Reduction

**DOI:** 10.3390/antiox13080960

**Published:** 2024-08-07

**Authors:** Beata Szlachta, Anna Birková, Beáta Čižmárová, Anna Głogowska-Gruszka, Paulina Zalejska-Fiolka, Maria Dydoń, Jolanta Zalejska-Fiolka

**Affiliations:** 1 Department of Biochemistry, Faculty of Medical Science, Zabrze Medical University of Silesia, 40-055 Katowice, Poland; b.szlachta@uk-brandenburg.de (B.S.); aglogowska@sum.edu.pl (A.G.-G.); s91883@365.sum.edu.pl (P.Z.-F.); m.dydon@nzozkruszyna.pl (M.D.); jzalejskafiolka@sum.edu.pl (J.Z.-F.); 2 Department of Medical and Clinical Biochemistry, Pavol Jozef Šafárik University, 040 11 Košice, Slovakia; beata.cizmarova@upjs.sk

**Keywords:** bioimpedance, weight loss, visceral fat loss, total body water loss, skeletal muscle mass loss, erythrocyte antioxidant enzymes, lipofuscin, malondialdehyde, glycemia

## Abstract

Background: This study aimed to investigate the impact of reductions in various body mass components on the erythrocyte oxidative status and glycemic state of people with obesity (PWO). Methods: A total of 53 PWO followed a six-month individualized low-calorie diet with exercise, during which anthropometric, biochemical, and oxidative parameters were measured. The participants were divided into groups based on weight (W), visceral fat area (VFA), total body water (TBW), and skeletal muscle mass (SMM) losses, as well as normoglycemia (NG) and hyperglycemia (HG). Results: Weight reduction normalized glycemia and influenced erythrocyte enzyme activity. Regardless of the tissue type lost (VFA, TBW, or SMM), glutathione peroxidase activity decreased in all groups, accompanied by an increase in glutathione reductase activity. Lipofuscin (LPS) and malondialdehyde (MDA) concentrations decreased regardless of the type of tissue lost. The α-/γ-tocopherol ratio increased in those losing >10% body weight, >15% VFA, and >5% TBW. In the NG group, compared to the HG group, there was a decrease in glutathione peroxidase and an increase in glutathione reductase, with these changes being stronger in the HG group. The LPS and MDA concentrations decreased in both groups. Significant correlations were observed between glucose reduction and changes in catalase, retinol, and α-tocopherol, as well as between VFA reduction and changes in vitamin E, L-LPS, and the activities of L-GR and L-GST. Conclusions: This analysis highlights the complex interactions between glucose metabolism, oxidative state, and erythrocyte membrane integrity, crucial for understanding diabetes and its management. This study shows the significant metabolic adaptability of erythrocytes in response to systemic changes induced by obesity and hyperglycemia, suggesting potential therapeutic targets to improve metabolic health in obese individuals.

## 1. Introduction

Obesity is a serious, chronic, complex disease that affects both children and adults. It is considered as a global health problem because its incidence is increasing worldwide. The prevalence of obesity has nearly tripled since 1975. Globally, in 2016, 39% of adults (aged 18 and over) were overweight and 13% were obese, while in 2020, 39 million children under the age of 5 were overweight or obese [1]. Eurostat reports that obesity rates, as well as problems with weight, are rapidly rising in most EU member states. In 2019, it was estimated that 52.7% of the adult population in the EU was overweight [2]. Obesity is associated with many serious conditions, such as cardiovascular diseases, diabetes mellitus, musculoskeletal diseases, certain types of cancer, liver dysfunction, dementia, sleep disorders, asthma, and infertility, while it is also associated with a reduction in life expectancy [3,4,5,6]. 

There is increasing evidence suggesting that oxidative stress plays an important role in the pathogenesis of obesity and its associated diseases. Also, a significantly positive correlation between BMI and biomarkers of oxidative stress has been observed [6]. In obese people, increased values of oxidative stress markers in plasma, serum, and urine have been observed, namely malondialdehyde, 8-epi-prostaglandin F2α, F-2 isoprostanes, thiobarbituric acid reactive species, and protein carbonyls [6,7]. 

In blood, erythrocytes (red blood cells, RBCs) are a promising model for the study of oxidative stress in the organism, as they are exposed to shear stress induced by blood flow, as well as oxidative stress in the circulation of blood. RBCs have an unparalleled organelle-free structure and unique biochemical and biophysical properties that define their functionality. Their lipid and protein composition changes during their lifetime, and specifically, these changes can be observed at the level of an RBC’s plasma membrane, consisting of 19.5% (*w*/*w*) water, 39.5% proteins, 35.1% lipids, and 5.8% carbohydrates [8]. Thanks to the simple isolation of erythrocytes and their membranes, as well as -omics approaches, there is a wealth of exact knowledge about the composition of molecules in erythrocytes. Detailed lipidomic analyses of RBC membranes point to a relation between membrane lipid composition and the rheological behavior of RBCs in the microvascular network [9]. Recently, Bryk et al. conducted a proteome analysis of RBCs to better elucidate their specific structure. In this study, the authors identified around 2500 proteins within RBCs. Among the 20 most abundant proteins in whole RBCs are chaperones and proteins mainly involved in the transport of gasses, cell antioxidant defense, membrane transport, and the major metabolic pathways in RBCs, which influence the glycolysis and motility of the cells [10]. 

In many pathological conditions (e.g., diabetes, cardiovascular diseases, and hemoglobinopathies) and aging, there are changes in RBCs’ biochemical and biophysical properties, and such changes can be used as markers of pathology in various diseases [11]. Oxidative stress, formed by excess reactive oxygen species (ROS) and eliminated by the action of enzymatic and non-enzymatic antioxidants in the human body, can also influence the structure and function of RBCs. RBCs contain antioxidant systems that fundamentally contribute to the function and integrity of these cells and maintain redox regulation in the body [12,13]. The antioxidant system of RBCs includes antioxidant enzymes, namely superoxide dismutase (SOD; RBCs contain Cu/Zn SOD), glutathione peroxidase (GPX), glutathione reductase (GR), catalase (KAT), peroxidases (Prx1, Prx2, and Prx6), and thioredoxin reductase (TR), and tripeptide glutathione (GSH) is considered as the main intracellular low-molecular-weight antioxidant. Other non-enzymatic antioxidants found in RBCs include vitamin C and vitamin E [14]. In obese individuals, there is evidence of reduced levels of antioxidant enzymes in RBCs (SOD, CAT, and GPX). Decreased levels of vitamin A, vitamin C, vitamin E, and β-carotene have also been observed in people with obesity (PWO), thought to be related to greater oxidative damage. In a previous study, it was shown that supplementation with antioxidants led to a reduction in oxidative stress [6,15]. 

In our previous publication [16] on the effect of reductions in various body mass components on the serum oxidative, glycemic, and lipid parameters of PWO, we found different patterns of activity changes in SOD isoenzymes. Lower mitochondrial SOD activity correlated with greater muscle and water loss, contrasting with cytosolic isoenzyme activity, which was lower in those retaining muscle and water. We also found that glucose level influenced weight reduction and redox state. Hyperglycemia is associated with higher total SOD levels, while improved glycemia increases mitochondrial SOD activity. Overall, we concluded that weight reduction reduces cardiovascular disease risk, yet active antioxidant molecules are more abundant in hyperglycemic cases, increasing further after glycemic improvement and weight loss. This prior study suggested that body weight alone inadequately predicts patients’ glycemic and redox statuses.

The present study discusses erythrocyte oxidative status in PWO, including individuals with and without hyperglycemia, who underwent a weight-reduction program (WRP). We also examined oxidative state depending on the tissue reduced during weight loss (muscle, fat, and water).

## 2. Materials and Methods

### 2.1. Ethical Permission

The study was approved by the Bioethical Committee of the Medical University of Silesia (SUM), with the reference number KNW/0022/KB1/19/I/16 (10 May 2016). The study was performed in accordance with the standards of the Declaration of Helsinki. The patients were informed about the procedure and potential risks before they were asked to participate. Written informed consent to participate in the study and publish the research results was obtained from all patients before the study started. Patients’ data were protected by a RODO statement (General Data Protection Regulation). To avoid bias, all samples were anonymized and numbered.

### 2.2. Study Population 

This study was based on reports indicating that body mass reduction can ameliorate metabolic impairment. It was a prospective, single-center study. 

The study inclusion criteria included the following: body mass index (BMI) > 30 kg/m^2^, no treatment affecting lipid and glucose metabolism, and informed consent to participate in the study. The study exclusion criteria included the following: lack of consent to participate in the study; disturbances of consciousness; severe hepatic, renal, respiratory, or circulatory insufficiency; chronic alcohol abuse; pregnancy; treatment-resistant depression; history of a serious nervous system injury; presence of an implanted cardiac pacemaker; and/or treatment affecting lipid and glucose metabolism.

A total of 53 people with obesity (PWO) (37 women and 16 men) were enrolled into a 6-month weight-reduction program (WRP), which consisted of a balanced, individualized, low-calorie diet, moderate exercise, and health education. They were monitored monthly during the follow-up visits, performed by a doctor and a dietitian. The patients were weighed, motivated to continue participating in the program, and received support in the form of training on how to calculate calorie intake and assess the quality of food products. Weight reduction was monitored until the achievement of a healthy body weight or a reduction in initial weight of 5–15%. 

### 2.3. Dietary and Physical Activity Intervention 

As stated, the patients were enrolled into a weight-reduction program (WRP), which was based on reports showing that weight loss improves lipid and glucose levels. Prior to the treatment program, all patients followed a poor-quality diet that was high in saturated fats, salt, simple sugars, refined grains, and preservatives, with a low intake of antioxidant-rich fruits and vegetables and minimal physical activity. During the first visit, after a medical examination and the completion of a questionnaire (which included history of obesity, health problems, food preferences, and physical activity), we determined each patient’s basal metabolic rate (BMR), comprehensive metabolic rate (CPM), and daily energy deficit (DDE) in kcal per day. We recommended a slight calorie restriction, calculated as 15% of energy based on CPM. Each patient received personalized instructions based on recommendations from the Polish National Food and Nutrition Institute, including the provision of resources such as a seven-day menu and a list of products, along with their glycemic index (IG) values, that could ensure sufficient water and vegetable/fruit intake, as outlined in Table 1 below.

The patients were advised to increase their vegetable consumption to 500 g per day and their fruit consumption to 200–300 g per day (only fruit until 4–5 p.m.) and to have five meals per day that were appropriate in terms of meal composition and caloric value. In addition to following a proper diet, the patients were encouraged to engage in moderate physical exercise for at least 40 min three times a week. The patients were also advised to only consume snacks with low or medium glycemic index (IG) values, if needed.

During the follow-up visits, which occurred every month, the patients were weighed, and their waist and hip circumferences were measured. Weight-loss results, as well as the quantity and composition of their diet, were recorded continuously. The patients were motivated to keep partaking in the WRP and were provided with support in the form of training on how to calculate calorie intake and assess the quality of food products.

Weight reduction was monitored until patients reached a healthy body weight or achieved a 5–15% reduction in their initial weight, which typically took six months.

### 2.4. Dividing Patients into Groups

No significant differences in glucose levels, BMI, VFA, or BFM (body fat mass) were observed between the male and female participants. Due to the similarity in responses, data from both sexes were analyzed collectively. The participants were divided into groups based on weight loss (WL), visceral fat area (VFA) loss, total body water (TBW) loss, and skeletal muscle mass (SMM) loss, with cut-off lines for each category as follows:
GroupSubgroup designationWeight loss (WL)WL < 10%WL > 10%Visceral fat area loss (VFAL)VFA < 15%VFA > 15%Total body water loss (TBWL)TBW < 5%TBW > 5%Skeletal muscle mass loss (SMML)SMM < 5%SMM > 5%

Weight reduction was monitored until patients reached a healthy body weight or achieved a 5–15% reduction in their initial weight, which typically took six months. Hence, we used a breakdown for PWO based on whether they achieved a weight reduction of less or more than 15% [17]. The ranges applied for VFA, SMM, and TW were based on the obtained research results and statistical analyses.

The participants were also divided into normo- (NG) and hyperglycemic (HG) groups based on the initial fasting glucose concentration.

### 2.5. Biochemical Assessment 

Fasting blood samples were collected at the Metabolic Clinic in Miasteczko Śląskie, Poland, from the cubital vein using clotting tubes to obtain serum (9 mL) and clot activator tubes (8 mL) to obtain plasma and erythrocytes. After centrifugation (10 min, 3000 rpm, 4 °C), the plasma was aliquoted, and 10% hemolysate was prepared in re-distilled water. All samples were stored at −80 °C at the Department of Biochemistry, Faculty of Medical Science, Medical University of Silesia, Katowice, Poland. To avoid bias, all samples were anonymized and numbered. Following good laboratory practices, biochemical markers were assessed during the first and last visits.

Glucose and insulin concentrations were determined in serum. Glucose (Glc) was measured using a BS-200E biochemical analyzer (Mindray, Shenzhen, China) and Alpha Diagnostics reagents (San Antonio, TX, USA). Insulin was determined using INS-IRMA kits (KIP1251-KIP1254, DIA Source Immuno Assays S.A., Louvain, Belgium). The repeatability values (within-run precision coefficients of variation) for glucose and insulin were 0.6% and 4.1%, respectively. The reproducibility values (between-run precision coefficients of variation) for the above-mentioned parameters were 1.6% and 7.7%, respectively. The HOMA-IR coefficient was calculated as follows:

[fasting insulin concentration [µIU/mL] × fasting glucose concentration [mg/dL])]/405.

### 2.6. Oxidative Status Parameters

Analysis of oxidative status parameters was performed in 10% erythrocyte hemolysate. Hemoglobin concentration was determined using the Drabkin method [18]. Analyses of the activity of L-SOD, L-KAT, L-GPX, L-GR, L-GST, L-LPS, and L-MDA were conducted using a PerkinElmer automated analyzer (PerkinElmer, Waltham, MA, USA). The activity of superoxide dismutase (L-SOD) was assessed using the Oyanagui method [19] and expressed in nitric units NU/mgHb. Catalase (L-KAT) activity was measured using the method of Johansson and Borg [20] and expressed as kIU/g Hb. Glutathione peroxidase (L-GPX) activity was measured using the kinetic method of Paglia and Valentine [21] and expressed as IU/g Hb. The activity of glutathione reductase (L-GR) was measured according to Richterich [22] and expressed as IU/g Hb. The activity of glutathione S-transferase (L-GST) was measured according to the kinetic method of Habig and Jakoby [23] and expressed as IU/g Hb. 

Lipofuscin (L-LPS) concentration was determined according to Jain [24] and is expressed as RF (relative fluorescence lipid extract)/gHb. Malondialdehyde (L-MDA) concentration was measured fluorometrically according to the methodology of Ohkawa, Ohishi, and Yagi [25] and expressed in µmol/gHb. 

Assessments of α-tocopherol, γ-tocopherol, and retinol concentrations were conducted in plasma using the HPLC (KNAUER HPLC equipment) assay outlined by Lim C.K. [26]. HPLC conditions: KNAUER HPLC equipment, HPLC pump 64, EUROCHROM 2000 for data analysis. Column: PS Spheribond 80-5 ODS2, 125 × 4.6 mm with pre-column. Mobile phase: 4% (*v*/*v*) water in methanol (POCH Gliwice, Poland, no 621991154). Flow rate: 1 [mL/min]; analysis time: 20 min; temp.: 40 °C. Fluorescence detector: Shimadzu RF-10Axl, wavelength absorbance for α-tocopherol ext. 325 nm em. 470 nm; for retinol ext. 292 nm em. 330 nm.

### 2.7. Body Mass Parameters

Body mass parameters were measured using the InBody S10 body composition analyzer (Biospace, Cerritos, CA, USA), which utilizes bioelectrical impedance spectroscopy. This method was selected for its efficiency and accuracy [27]. The InBody S10 has received ISO 9001:2015 and ISO 13485:2016 certification, as well as EN60601-1, EN60601-1-2, and CE MDD (Directive 93/42/EEC) medical certificates [28,29,30,31,32].

A standardized measurement protocol was implemented to obtain accurate bioimpedance results. This included recording age, sex, height, and body mass and conducting measurements at consistent time slots. Specific guidelines were followed, such as fasting for 3 h, abstaining from alcohol for 12 h, and avoiding medications that affect water balance. Subjects removed accessories, heavy clothing, and jewelry, and measurements were taken after standing upright for at least 5 min. Repeatability was assessed with 12 measurements on the same individual, resulting in coefficients of variation below 3% for all parameters.

### 2.8. Statistical Analysis 

The results presented herein represent results from before and after the 6-month weight-reduction program. The calculated differences between these values are expressed as delta (Δ). All results are shown as the mean ± SD. Statistical analysis was performed with SPSS Statistics 22 (IBM, Armonk, NY, USA). The Kolmogorov–Smirnov test was used for normality testing. A paired *t*-test was used to determine differences in clinical parameters between values within groups before and after the dietary intervention. An unpaired *t*-test was used to determine differences in clinical parameters between groups, and assumptions of equal variances were tested using Levene’s test for equality of variances. The Pearson correlation coefficient was used to express the strength and direction of linear relationships between two parameters. Statistical significance was assumed at a *p*-value of <0.05.

## 3. Results

Table 2 shows a comparison of the determined parameters for the entire cohort of patients (without dividing them into groups) before and after the WRP. In the whole group of PWO (Table 2), weight reduction did not lead to significant changes in the activity of L-SOD and L-KAT or in the concentrations of retinol, alpha-tocopherol, and gamma-tocopherol. At the same time, increases in L-GST and L-GR activity and the α/γ-tocopherol ratio were observed. In contrast, the concentrations of glucose, MDA, and LPS decreased, and the insulin resistance index (HOMA-IR) and the activity of L-GPX were significantly reduced. In terms of body mass composition, a significant reduction was observed in all analyzed parameters (weight, VFA, TBW, and SMM) during the experiment.

### 3.1. Oxidative Stress Parameter Levels Related to Specific Compartment Loss

The results regarding the selected oxidative stress parameters for all the study groups are presented in Table 3. 

The activity of L-SOD decreased significantly in group WL > 10%. A trend showing a decrease in L-SOD was also visible in groups SMM > 5% and TBW > 5%. The activity of L-GPX and the concentration of L-LPS and L-MDA decreased significantly in all study groups. The activity of L-GST increased significantly only in VFA > 15%, TBW < 5%, and SMM < 5%. The activity of L-GR increased significantly in all study groups. There were no changes in L-KAT activity in all studied groups, with the exception of WL > 10%. In this group, the L-KAT activity increased significantly after the WRP. The concentration of α-tocopherol increased significantly in the SMM < 5% group, while the concentration of γ-tocopherol decreased significantly in the WL > 10%, TBW > 5%, and SMM > 5% groups. The ratio of α-tocopherol to γ-tocopherol increased in WL > 10%, VFA > 15%, and TBW > 5%.

Despite the higher L-SOD activity before body mass reduction in the TBW < 5% group, the activity of this parameter was significantly lower after weight reduction compared to the TBW > 5% group. Also, in the SMM > 5% group, L-SOD activity was significantly lower compared to the SMM < 5% group after weight reduction. L-GPX activity was statistically significantly higher in the TBW > 5% and SMM > 5% groups before weight reduction compared to the TBW < 5% and SMM < 5% groups, respectively. The activity of L-GPX was also higher in the SMM > 5% group after reduction compared to the SMM < 5% group. L-GST activity was significantly lower in the SMM > 5% group before weight reduction and in the VFA > 15% group compared to the VFA < 15% group after weight reduction. However, L-LPS decreased in all groups, with the lowest values being found for patients with greater SMM loss and lower VFA loss. The concentration of γ-tocopherol was lower in WL > 10% than WL < 10% after the WRP, but the ratio of α-/γ-tocopherol was higher in WL > 10% than WL < 10% after the body mass reduction program.

### 3.2. Effects of Glucose Adjustment and VFA Reduction on Antioxidant Properties of Erythrocytes

Correlation analysis revealed few relations between the changes in the measured parameters. The results are shown as delta:

∆ = parameter before study (WRP) (X_1) − parameter at the end of the study (WRP) (X_2)

**Figure 1:**
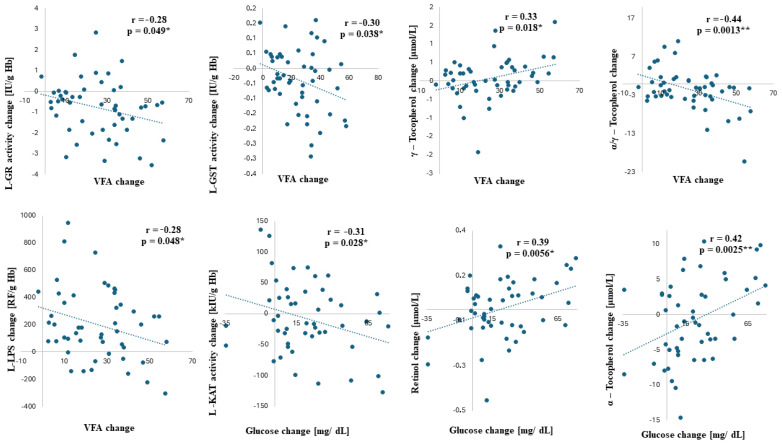
The correlations between selected parameters. Pearson’s coefficient r expresses the strength of the correlation. The correlation is significant if *p* < 0.05 (*) and *p* < 0.005 (**). **Legend**: VFA—visceral fat area; L-GST—hemolysate glutathione transferase; L-GR—hemolysate glutathione reductase; L-KAT—hemolysate catalase; and L-LPS—hemolysate lipofuscin.

The most significant (Figure 1) correlations were between glucose reduction vs. catalase; between retinol and α-tocopherol changes; and between VFA reduction vs. vitamin E, L-LPS, L-GR, and L-GST activity changes. With a higher glucose decrease after the WRP, there was a higher increase in L-KAT activity (r = −0.31; *p* = 0.028). The opposite relation was observed between glucose decrease and retinol and α-tocopherol changes, as the glucose decrease correlated with retinol (r = 0.39; *p* = 0.0056) and α-tocopherol decreases (r = 0.42; *p* = 0.0025). The WRP-related decrease in VFA correlates negatively with the decreases in LPS (r = −0.28; *p* = 0.048*) and γ-tocopherol (r = 0.33; *p* = 0.018*) and with the increase in the α/γ-tocopherol ratio (r = −0.44; *p* = 0.0013). Also, the higher the VFA reduction, the higher the increases in L-GR (r^2^ = −0.28; *p* = 0.049) and L-GST activity (r = −0.30; *p* = 0.038).

### 3.3. Results of Studied Parameters in Normo- and Hyperglycemia Patients

Considering the correlations between glucose and some anti- and pro-oxidant parameters, we decided to show the changes in oxidative status in the normo- and hyperglycemic groups (Table 4). There were no differences in loss of weight, VFA, TBW, or SMM, or in L-KAT activity and retinol concentration between the normo- (NG) and hyperglycemic (HG) groups. There was no change in L-SOD activity in either group, but L-SOD activity was lower in the HG group after the WRP in comparison to the NG group. The L-GPX activity was lower in the NG group before the WRP and decreased in both groups after the WRP, with no differences observed between the groups. The L-GST activity decreased in the NG group after the WRP, and the activity was lower in the HG group after the program. There were no differences between the groups in L-GR activity, and the activity of L-GR decreased in both groups after the WRP. The L-LPS concentration was lower in the HG group both before and after the WRP, and it decreased in both study groups after the WRP. The L-MDA concentration decreased in both groups and was lower in the HG group after the WRP. There were no differences in the α- and γ-tocopherol concentrations and the α/γ-tocopherol ratio before or after the WRP between the NG and HG groups. Simultaneously, the concentration of α-tocopherol decreased in the NG group, while γ-tocopherol and the α/γ-tocopherol ratio decreased in the HG group after the WRP.

## 4. Discussion

Obesity, often accompanied by metabolic disorders such as insulin resistance and hyperglycemia, significantly impacts cellular functions across the body, including RBCs. Some changes mimic the alterations induced by aging, but others have the opposite effect, possibly counteracting the adaptation of the organism to senescence [33]. Obesity induces a state of chronic oxidative stress, affecting erythrocytes’ functionality by altering glucose metabolism pathways and increasing the production of ROS. However, existing human and animal studies show controversial results regarding obesity and antioxidant enzymes. Some studies have shown that antioxidant enzymes increase in obesity, but there are studies that found no significant difference in antioxidant enzyme concentrations in obese individuals [34]. The present study involved only people with obesity (PWO). We examined their responses after weight reduction from a few perspectives. We explored erythrocyte responses to oxidative stress in obese subjects undergoing weight loss, highlighting the differences between those with a normoglycemic state (NG) and those with a hyperglycemic state (HG) before the WRP. This study also describes the effect on RBC redox state in relation to different types of tissue loss during a weight-reduction program (WRP). 

**Figure 2:**
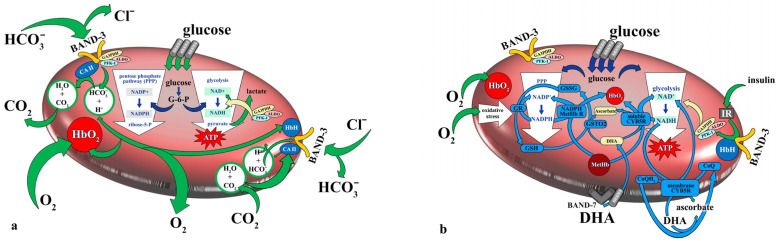
(**a**,**b**). Mutual interactions between glucose metabolism, oxidative state, and cell membrane in mature RBCs. RBCs are important glucose-consuming cells, and there are few metabolic pathways involved in glucose consumption. Glycolysis is a producer of NADH, lactate for other cells, and is the only source of ATP. The pentose phosphate pathway (PPP) is a source of NADPH, vital for the regeneration of glutathione. Under physiological conditions, about 7% of glucose is used in the PPP. Increased glucose influx into RBCs can be a reason for PPP increase. Band-3, the anion exchanger of HCO_3_^−^/Cl^−^, binds carbonic anhydrase II (CA II) and deoxygenated hemoglobin HbH. After hemoglobin oxygenation (HbO_2_), glycolytic enzymes are bound to band-3 instead of HbH. Besides glucose-6-phosphate dehydrogenase activity, this is the second regulatory mechanism which helps to shift glucose-6-phosphate to glycolysis (in the case of HbH prevalence) or PPP (in the case of HbO_2_ prevalence). Insulin-independent glucose transporter 1 (GLUT1) interacts with various membrane proteins, such as band-7, dematin, adducin, and band-3. GLUT1 is also able to transport dehydroascorbic acid (DHA). Band-7’s interaction with GLUT1 causes a change in the substrate preference of the transported molecule from glucose to DHA. Ascorbate’s involvement in antioxidant activity involves cooperation with both PPP and glycolysis. In hyperglycemic individuals, there can be a reduced level of ascorbate in RBCs, resulting from the competitive effect of DHA and glucose or due to the changed expression of GLUT1. The actin cytoskeleton complexes around GLUT1 may be chemically modified in diabetic individuals’ RBCs. The dynamics of the cytoskeleton/actin can regulate glucose uptake through GLUT1. The human RBC membrane also contains the insulin receptor (IR). Insulin activates PFK (6-phosphofructo-1-kinase) via phosphorylation, causing it to detach from band-3 and leading to the redistribution of the enzyme within RBCs, thus supporting glycolysis. The enzymes glutathione reductase (GR), glutathione-S-transferase (GSTO2), NADPH methemoglobine reductase (NADPH MetHb R), and cytochrome B5 reductase (CYB5R) are closely dependent on metabolic pathways [35,36,37,38,39,40,41,42,43,44,45,46,47,48,49].

The interactions between glucose metabolism, oxidative state, and the integrity of erythrocyte cell membranes are highly complex. The most important processes involving RBC (Figure 2a) and their associations to redox state (Figure 2b) are shown in Figure 2. 

The glucose uptake rate, enzyme activity, and production and utilization of intermediate metabolites and ATP in the erythrocytes of obese patients with hyperglycemia can be altered [46,50]. Insulin resistance, a common feature of obesity, influences RBC function by modulating GLUT1 expression and glucose uptake. On one hand, increased glucose metabolism in RBCs helps to consume excess glucose from the blood and thus reduces the formation of glycated end-products (AGEs) in the serum. On the other hand, it intensifies glycation processes in RBCs, leading to oxidative stress and causing lipid and protein oxidation, resulting in their denaturation and changes in the structure and function of RBCs. These changes lead to a progressive decline in RBC deformability, associated with increased fragility and shortened lifespan [48,51,52]. Insulin-mediated glucose metabolism in erythrocytes highlights the complex interaction between insulin sensitivity and RBC health.

RBC defensive mechanisms are crucial in managing oxidative stress in erythrocytes and may have significant implications for the treatment and management of diabetes, where controlling hyperglycemia and protecting against lipid peroxidation can significantly impact the reduction in complications [53,54]. 

In response to lipid peroxidation, RBCs utilize several antioxidant enzymes that help protect the cells from damage. Glutathione peroxidase (GPX) is a key enzyme that reduces lipid peroxides to alcohols, using reduced glutathione (GSH) as an electron donor. The byproduct of this reaction is oxidized glutathione (GSSG), which is then reduced back to GSH by glutathione reductase (GR) in the presence of NADPH, ensuring the continuation of the antioxidant cycle. GST also plays a critical role in RBC protection. The peroxidase activity of GST, crucial for breaking down lipid peroxides, not only relies on selenium but also requires the presence of GSH. GSH’s coupling reaction with electrophilic products of lipid peroxidation leads to the neutralization of harmful aldehydes and other peroxidation products. Thus, GST helps reduce toxicity and improve the solubility of these products in water, facilitating their elimination from the cell [46,50]. In the present study, regardless of the type of tissue loss, WRP led to a significant reduction in L-GPX and a significant increase in L-GR, without significant changes in L-SOD or L-CAT, although more drastic weight loss was accompanied with a decrease in L-SOD and an increase in L-CAT.

Weight loss is usually not only accompanied by losses of fat but also reductions in other aspects, including body water and muscle mass. However, losing lean body mass has a number of negative health consequences, which is why a lot of research is focused on strategies to maintain muscle mass during weight loss, including the use of a wide range of nutritional supplements [55]. In our previous publications, we showed that obese patients with persistent hyperglycemia are resistant to fat tissue reductions even after an extensive WRP based on balanced nutrition and exercise [56]. Moreover, we showed different lipid metabolism responses depending on the extent of muscle loss in PWO. Greater muscle loss was associated with a decrease in triacylglycerols, reflecting the loss of fat stores, but smaller muscle losses were linked to an increase in beneficial HDL-cholesterol. In addition, different patterns of changes in superoxide dismutase (SOD) isoenzyme activity were observed. Patients with lower mitochondrial MnSOD activity in serum at the outset were more likely to lose more non-fat tissue, and mitochondrial MnSOD activity in serum significantly increased after glycemia improvement. On the contrary, lower cytosolic CuZnSOD activity in serum at the outset was found to be related to smaller losses of non-fat tissues and significantly increased after the WRP in normoglycemic patients, but this did not occur in the hyperglycemia patients [16]. Isoenzyme SOD activity responses may also vary in different skeletal muscle fiber types upon stimuli, as has been described, for example, in aging [57]. Circulating cytosolic SOD, derived from both hemolysis and peripheral tissues as a result of cellular secretion [58], is the major SOD isoenzyme in RBCs, and in this study, it did not experience any changes in general during the WRP. However, patients losing more muscle finished with significantly lower L-SOD values compared to patients with less muscle loss after the WRP, highlighting the importance of preventing muscle loss due to the impact on RBCs’ antioxidant defense.

The mechanisms underlying muscle mass reduction during weight loss are not fully understood. Evidence indicates that a short-term (up to 21 days) calorie restriction (about 35% energy deficit/day) can decrease the postprandial rate of muscle protein synthesis and reduce the basal rate of muscle protein synthesis [59]. Contrastingly, in another study, it was indicated that a prolonged moderate calorie restriction and 5–10% weight loss increased the rate of muscle protein synthesis [60]. Overall, muscle mass reduction during a prolonged moderate calorie restriction is believed to be mediated by increased muscle proteolysis rather than suppressed muscle protein synthesis, and it is viewed as an undesirable effect. It appears that increased physical activity during weight loss may protect against this unfavorable process [61]. The results of the current study indicate that other factors that affect the type of tissue being reduced during weight loss should also be taken into account. One of these factors could be hyperglycemia state.

Also, glycation was involved in the change in L-SOD during the WRP. In this study, before the WRP, there was no significant difference between SOD levels in the NG and HG patients, which is in accordance with Turpin et al. (2020) [62], who showed no differences in SOD levels in glycated RBCs compared to non-glycated RBCs. However, after the WRP, in NG PWO, there was a slight increase in L-SOD activity (non-significant), and in HG PWO, there was a slight decrease in L-SOD activity (non-significant), finally leading to significantly lower levels in the HG group compared to the NG group, thus pointing to the importance of glycemia in obesity as an important factor negatively affecting WRPs. Turpin et al. (2020) [62] also showed no difference in glycated RBCs compared to non-glycated RBCs in terms of catalase levels. Similarly, in the present study, no difference was detected in normo- vs. hyperglycemic PWO before the WRP. Regarding other enzymes, Turpin et al. also measured peroxidase activity, which was decreased in glycated RBCs, differing from what we found in our study. However, these other authors measured total peroxidase activity and focused on the relation to diabetes regardless of obesity.

Following the WRP, the RBCs of the NG individuals showed improved antioxidant defense, indicated by a decrease in the activity of glutathione peroxidase (L-GPX) and increased activities of glutathione reductase (L-GR) and, notably, glutathione S-transferase (L-GST). This was also observed in those who lost more than 15% of their VFA. This contrasts with the results for the HG individuals, for which only the L-GPx and L-GR activities were changed after the WRP. Interestingly, the correlation analysis showed that glucose correction is related to a reduction in lipid-soluble antioxidant vitamins and an increase in the activity of L-KAT, which, before the diet program, was lower in the HG group (though not significantly so) compared to the NG group. L-KAT and L-GPX act on the same substrate, and according to Gaetani et al. [63,64], both are equally active in the detoxification of hydrogen peroxide, but L-GPX was, contrary to catalase, significantly higher in terms of activity in the HG group before the WRP compared to the NG group. This inverse relation between L-KAT and L-GPX in the HG group is probably related to the action of NADPH, which is essential for glutathione regeneration but also directly binds L-KAT and protects the molecule against oxidative damage and denaturation [64,65]. The higher the consumption of NADPH for glutathione reduction, necessary for L-GPX activity, the less NADPH available to bind and protect L-KAT. Another explanation is that L-GPX shows a higher affinity for hydrogen peroxide than L-KAT and therefore plays a more important role in most physiological situations when the amount of hydrogen peroxide produced is not too high. Insufficient L-KAT activity is thus compensated for by an increase in L-GPX activity, and conversely, reduced peroxidase activity is compensated for by an increase in L-KAT activity [65]. Ramezanipour et al. (2014) [66] studied the effect of weight reduction on antioxidant enzymes in a group of 30 obese women, specifically focusing on values from the beginning of the study and after a 3-month intervention. In terms of their results, the glutathione reductase and catalase activities showed a significant increase after weight reduction, but no significant changes were seen in the superoxide dismutase and glutathione peroxidase activities, as partially corroborated by our study. 

**Figure 3:**
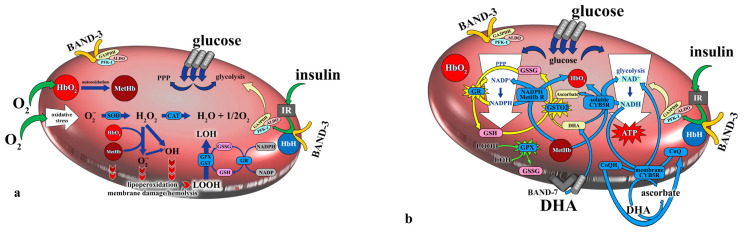
(**a**,**b**). The formation of ROS in RBCs and their processing and scavenging by enzymes. The most important enzymes are superoxide dismutase (SOD), catalase (CAT), glutathione peroxidase (GPX), and glutathione transferase (GST). Glutathione reductase (GR) is vital for glutathione’s recovery from the oxidized form (GSSG) to the reduced form (GSH). Glutathione-dependent enzymes are involved in protecting against lipid peroxidation via reducing lipoperoxide (LOOH) to alcohol (LOH). Additionally, GR is dependent on the availability of NADPH from the PPP (pentose phosphate pathway). Weight reduction itself results in a decrease in GPX activity and an increase in GR activity regardless of the type of tissue loss or glycemia level. A different pattern was found for GST, which increased only in the normoglycemia patients and did not experience change in the hyperglycemia patients. Water-soluble ascorbate seems to be a factor involved in GST changes in normo- and hyperglycemia patients, and this could also be connected to the type of tissue loss, as GST only increased in patients who lost less than 5% of their total body and skeletal muscle mass.

Moreover, generally, glutathione-related enzyme activities showed different patterns between groups. In the NG group, L-GPX significantly decreased, and L-GR and L-GST significantly increased, contrary to the HG group, in which there was no change in L-GST activity. Additionally, correlation analysis showed that the changes in L-GR and L-GST were not related to glucose improvement, but their increased activities in RBCs are associated with VFA reduction. The increase in L-GST activity observed in the NG obese individuals (Figure 3) indicates the potential for better antioxidant protection for the normoglycemic subjects after weight reduction and highlights the different metabolic adaptations of RBCs to weight loss in obese patients with normo- and hyperglycemia. Our results also indirectly point to an improvement in NADPH availability after glycemia correction.

Regarding vitamins, the most significant changes were present in patients who lost more weight, more VFA, and more TBW. We also found different vitamin E levels in normo- vs. hyperglycemia patients. Both vitamins are proven to have significant effects on RBCs. Vitamin A is involved in strengthening RBC membrane integrity, but with higher doses, it may exert an undesirable hemolytic effect [67], while vitamin E cooperates with vitamin C in RBC protection [68]. Both vitamin A and E exert antioxidant properties, but while vitamin E is a direct antioxidant preventing lipid peroxidation, vitamin A acts indirectly via the regulation of the transcription of a number of genes involved in mediating the body’s canonical antioxidant responses [68]. Although our study was without significant differences in retinol levels, retinol levels decrease with a decrease in glucose, thus implying an inter-relationship. This correlation between retinol and glucose levels was also described by Gök et al. [69] in their work studying antioxidant enzymes and vitamins in women with uterine myoma and endometrial cancer. Different levels of both vitamins A and E, relative to the control group, were also described by Darenskaya et al. [70], who reported lower values of retinol and α-tocopherol in those with diabetic nephropathy (both stage 1 and 2). They focused on men with T1DM, and both α-tocopherol and retinol show high sensitivity (ROC and AUC) in the earlier stage of diabetic nephropathy but not in the later stage. 

The results regarding L-LPS are also interesting. LPS in RBCs was significantly lower in the HG group both before the WRP and after the WRP. However, a decrease in L-LPS does not correlate with a decrease in glucose, but there is a relation between VFA and L-LPS, which is inverse and shows that the higher the decrease in VFA, the lower the decrease in LPS in RBCs. Similar results were also found in our compartment loss analysis, as L-LPS decreased the most in those who lost less weight, less VFA, more TBW, and more muscle. In RBCs, much of the lipid oxidation is catalyzed by HbO_2_ and is inhibited when hemoglobin is present as MetHb [71]. Also, hypercapnia exerts a protective effect against LPS formation [72]. Thus, in the HG group, the lower content of oxygenated hemoglobin was probably the cause of the low LPS levels.

## 5. Conclusions

This comprehensive analysis underscores the complex interactions between glucose metabolism, oxidative state, and erythrocyte membrane integrity, which are critical for understanding metabolic processes in diabetes and their management. This study highlights the significant metabolic adaptability of erythrocytes in response to systemic changes induced by obesity and hyperglycemia, suggesting avenues for future research to explore potential therapeutic targets in order to improve metabolic health in obese individuals.

## 6. Study Limitation

Despite the admission of 300 eligible patients to the program, compliance was met only by 53, which were included in the study. This was due to the following reasons: (a) the majority of the originally preselected patients appeared to be taking medications affecting glucose and lipid metabolism; (b) we experienced patient dropouts, as some patients who started the program stopped attending follow-up visits before the program was terminated; and (c) despite declaring they would do so, some patients did not follow the prescribed diet and/or physical activity regimen.

## Figures and Tables

**Table 1:** Composition of patients’ diet, designed according to the healthy nutrition recommendations of the Polish National Food and Nutrition Institute.

Nutrient	% of Total Energy Intake and Limitations	
Carbohydrates	45–65%	Limiting the intake of free sugars to less than 10% of total energy intake.
Fats	25–35%	Limiting the intake of saturated fatty acids to less than 10% of total energy intake and intake of 3–6% mono- and polyunsaturated fatty acids in the form of fish and vegetable oils.
Proteins	10–15%	Animal and vegetable sources.

**Table 2:** General description of the whole group of probands. Data are expressed as the mean ± SD. A *t*-test was used for a comparison of the parameters before and after weight-reduction program (WRP). The difference is significant if *p* < 0.05.

Parameter	Before WRP	After WRP	*p*
Weight	101.0 ± 21.0	90.7 ± 19.0	2.1*10^−13^
VFA	142.2 ± 34.3	115.5 ± 33.5	2.1*10^−15^
TBW	44.2 ± 9.5	41.2 ± 8.7	6.4*10^−5^
SMM	33.8 ± 7.7	31.2 ± 6.9	**0.0002**
Glc	117.7 ± 38.7	90.6 ± 16.9	8.2*10^−6^
HOMA IR	3.70 ± 3.38	2.53 ± 2.16	**0.005**
L-SOD	179.4 ± 15.4	176.9.0 ± 18.2	0.49
L-GPX	61.91 ± 10.6	52.6 ± 15.7	4.9*10^−9^
L-GST	0.22 ± 0.11	0.26 ± 0.13	**0.015**
L-KAT	472.4 ± 75.3	483.6 ± 69.5	0.23
L-GR	8.41 ± 2.1	9.13 ± 1.9	**0.00011**
L-LPS	1100.9 ± 235.1	913.9 ± 234.4	8.2*10^−6^
L-MDA	0.39 ± 0.09	0.33 ± 0.05	5.1*10^−5^
Retinol	0.62 ± 0.16	0.62 ± 0.16	0.70
α-tocopherol	26.24 ± 7.55	26.92 ± 8.53	0.38
γ-tocopherol	1.62 ± 0.59	1.54 ± 0.57	0.30
Ratio α/γ tocopherol	17.40 ± 5.55	18.88 ± 7.44	**0.042**

Legend: WRP—weight-reduction program; weight [kg]; VFA—visceral fat area [cm^2^]; TBW—total body water [L]; SMM—skeletal muscle mass [kg]; HOMA-IR—homeostatic model assessment for insulin resistance; L-SOD—hemolysate superoxide dismutase [NU/mgHb]; L-GPX—hemolysate glutathione peroxidase [IU/gHb]; L-GST—hemolysate glutathione transferase [IU/gHb]; L-GR—hemolysate glutathione reductase [IU/gHb]; L-KAT—hemolysate catalase [kIU/gHb]; L-LPS—hemolysate lipofuscin [RF/gHb]; L-MDA—hemolysate malondialdehyde [µmol/gHb]; retinol [µM/L]; α-tocopherol [µM/L]; and γ-tocopherol [µM/L].

**Table 3:** Statistical analysis of selected anti- and pro-oxidative parameters in individual groups of people with obesity (PWO). Data are expressed as mean ± SD. For within-group (p (X_1 vs. X_2)) and between-group (#p) comparisons of the differences between measurements before and after the WRP a *t*-test was used.

Parameter	X	WEIGHT LOSS		VISCERAL FAT AREA LOSS		TOTAL BODY WATER LOSS		SKELETAL MUSCE MASS LOSS	
		WL < 10% N = 34	WL > 10% #p N = 19	VFA < 15% N = 21	VFA > 15% #p N = 31	TBW < 5% N = 27	TBW > 5% #p N = 20	SMM < 5% N = 24	SMM > 5% #p N = 24
L-SOD	1	177.7 ± 15.03	182.5 ± 15.4 ^#0.29^	178.2 ± 14.8	180.8 ± 15.9 ^#0.56^	179.4 ± 13.45	181.3 ± 17.8 ^#0.68^	180.91 ± 13.82	179.84 ± 16.68 ^#0.81^
	2	180.1 ± 19.8	171.2 ± 14.0 ^#0.09^	175.0 ± 20.1	176.8 ± 15.7 ^#0.72^	181.4 ± 16.68	171.8 ± 16.0 ^#0.06^	183.57 ± 17.37	171.07 ± 13.65 ^#0.009^
p (X_1 vs. X_2)		0.29	**0.041**	0.58	0.36	0.63	0.12	0.54	0.10
L-GPX	1	61.0 ± 11.1	63.6 ± 9.8 ^#0.41^	64.84 ± 10.44	59.76 ± 10.6 ^#0.10^	58.3 ± 10.22	64.04 ± 10.4 ^#0.07^	56.39 ± 9.34	65.67 ± 9.74 ^#0.002^
	2	51.7 ± 15.5	54.1 ± 16.3 ^#0.60^	57.47 ± 15.79	49.43 ± 15.2 ^#0.07^	49.92 ± 15.3	54.2 ± 17.8 ^#0.38^	47.92 ± 15.33	56.13 ± 16.36 ^#0.08^
p (X_1 vs. X_2)		8.2*10^−8^	**0.0066**	**0.0007**	6*10^−6^	**0.0002**	**0.0003**	**0.0003**	**0.0002**
L-GST	1	0.22 ± 0.11	0.21 ± 0.09 ^#0.82^	0.21 ± 0.11	0.22 ± 0.10 ^#0.78^	0.23 ± 0.12	0.21 ± 0.1 ^#0.45^	0.25 ± 0.12	0.19 ± 0.09 ^#0.08^
	2	0.25 ± 0.13	0.28 ± 0.14 ^#0.49^	0.21 ± 0.12	0.29 ± 0.13 ^#0.046^	0.28 ± 0.14	0.24 ± 0.14 ^#0.40^	0.29 ± 0.14	0.23 ± 1.13 ^#0.12^
p		0.10	0.08	0.97	**0.013**	**0.038**	0.40	**0.052**	0.26
L-KAT	1	495.5 ± 74.0	428.6 ± 57.2 ^#0.002^	469.8 ± 66.6	471.7 ± 82.0 ^#0.93^	469.2 ± 85.6	479.3 ± 69.6 ^#0.67^	484.1 ± 80.4	462.1 ± 75.2 ^#0.34^
	2	496.3 ± 69.8	461.5 ± 64.9 ^#0.08^	487.8 ± 63.8	476.2 ± 70.7 ^#0.55^	481.2 ± 65.8	484.8 ± 77.1 ^#0.86^	482.1 ± 61.9	481.0 ± 78.1 ^#0.96^
p (X_1 vs. X_2)		0.93	**0.047**	0.12	0.84	0.32	0.87	0.87	0.22
L-GR	1	8.77 ± 2.34	7.74 ± 1.35 ^#0.05^	8.24 ± 1.98	8.51 ± 2.24 ^#0.67^	8.71 ± 2.37	8.28 ± 1.67 ^#0.50^	8.80 ± 2.45	8.15 ± 1.65 ^#0.29^
	2	9.15 ± 2.21	9.11 ± 1.04 ^#0.93^	8.88 ± 2.04	9.28 ± 1.76 ^#0.47^	9.56 ± 1.94	8.89 ± 1.76 ^#0.24^	9.60 ± 2.03	8.82 ± 1.71 ^#0.16^
p (X_1 vs. X_2)		**0.048**	**0.0003**	**0.046**	**0.001**	**0.007**	**0.004**	**0.015**	**0.002**
L-LPS	1	1114.7 ± 257.9	1074.8 ± 188.7 ^#0.57^	1082.5 ± 301.8	1117.9 ± 182.5 ^#0.60^	1121.0 ± 241.5	1104.8 ± 195.5 ^#0.81^	1150.8 ± 234.1	1058.4 ± 214.3 ^#0.16^
	2	914.5 ± 249.4	913.0 ± 212.4 ^#0.98^	811.6 ± 216.4	980.1 ± 226.5 ^#0.01^	987.5 ± 219.7	865.5 ± 235.8 ^#0.08^	1026.8 ± 223.6	829.0 ± 204.8 ^#0.003^
p (X_1 vs. X_2)		**0.0005**	**0.0051**	**0.0007**	**0.002**	**0.02**	**0.0003**	**0.06**	1.85*10^−5^
L-MDA	1	0.39 ± 0.11	0.38 ± 0.05 ^#0.44^	0.37 ± 0.09	0.39 ± 0.10 ^#0.53^	0.38 ± 0.09	0.41 ± 0.10 ^#0.32^	0.39 ± 0.09	0.39 ± 0.10 ^#0.79^
	2	0.32 ± 0.05	0.34 ± 0.04 ^#0.12^	0.31 ± 0.05	0.34 ± 0.04 ^#0.015^	0.34 ± 0.04	0.33 ± 0.04 ^#0.43^	0.34 ± 0.04	0.32 ± 0.04 ^#0.22^
p (X_1 vs. X_2)		**0.0006**	**0.027**	**0.003**	**0.010**	**0.019**	**0.0096**	**0.023**	**0.008**
Retinol	1	0.62 ± 0.17	0.63 ± 0.15 ^#0.78^	0.67 ± 0.18	0.59 ± 0.15 ^#0.10^	0.60 ± 0.19	0.64 ± 0.15 ^#0.40^	0.58 ± 0.19	0.66 ± 0.13 ^#0.13^
	2	0.63 ± 0.15	0.60 ± 0.17 ^#0.56^	0.65 ± 0.17	0.59 ± 0.15 ^#0.24^	0.62 ± 0.16	0.60 ± 0.18 ^#0.66^	0.62 ± 0.17	0.60 ± 0.16 ^#0.73^
p (X_1 vs. X_2)		0.71	0.26	0.52	0.83	0.42	0.10	0.16	**0.030**
α-tocopherol	1	26.44 ± 8.53	26.59 ± 6.13 ^#0.94^	27.36 ± 8.40	24.63 ± 5.38 ^#0.20^	25.53 ± 7.63	26.75 ± 6.44 ^#0.58^	24.75 ± 7.11	27.12 ± 7.07 ^#0.26^
	2	27.33 ± 9.70	26.03 ± 6.28 ^#0.57^	27.52 ± 8.55	25.27 ± 6.19 ^#0.29^	27.17 ± 7.90	25.51 ± 7.15 ^#0.48^	27.33 ± 8.36	25.52 ± 6.48 ^#0.42^
p (X_1 vs. X_2)		0.23	0.65	0.90	0.54	0.16	0.28	**0.028**	0.16
γ-tocopherol	1	1.65 ± 0.64	1.55 ± 0.50 ^#0.53^	1.64 ± 0.68	1.55 ± 0.50 ^#0.61^	1.55 ± 0.60	1.73 ± 0.57 ^#0.33^	1.53 ± 0.57	1.72 ± 0.59 ^#0.29^
	2	1.68 ± 0.65	1.28 ± 0.26 ^#0.003^	1.62 ± 0.55	1.43 ± 0.54 ^#0.21^	1.62 ± 0.63	1.39 ± 0.37 ^#0.13^	1.64 ± 0.67	1.44 ± 0.38 ^#0.21^
p (X_1 vs. X_2)		0.78	**0.026**	0.90	0.26	0.55	**0.0016**	0.34	**0.019**
Ratio α-/γ- tocopherol	1	16.96 ± 5.26	18.35 ± 6.22 ^#0.43^	17.97 ± 5.45	17.06 ± 5.86 ^#0.58^	17.38 ± 4.91	16.98 ± 6.86 ^#0.82^	17.06 ± 4.71	17.19 ± 6.69 ^#0.94^
	2	17.23 ± 4.75	21.87 ± 10.43 ^#0.09^	17.57 ± 4.22	19.74 ± 9.27 ^#0.27^	18.01 ± 5.89	19.84 ± 10.2 ^#0.50^	18.03 ± 6.20	19.17 ± 9.43 ^#0.63^
p (X_1 vs. X_2)		0.72	**0.029**	0.66	**0.015**	0.56	**0.027**	0.39	0.10

**Legend:** X_1—before WRP; X_2—after WRP; weight [kg]; VFA—visceral fat area [cm^2^]; TBW—total body water [L]; SMM—skeletal muscle mass [kg]; L-SOD—hemolysate superoxide dismutase [NU/mgHb]; L-GPX—hemolysate glutathione peroxidase [IU/gHb]; L-GST—hemolysate glutathione transferase [IU/gHb]; L-GR—hemolysate glutathione reductase [IU/gHb]; L-KAT—hemolysate catalase [kIU/gHb]; L-LPS—hemolysate lipofuscin [RF/gHb]; and L-MDA—hemolysate malondialdehyde [µmol/gHb]; Retinol [µmol/L]; α-tocopherol [µmol/L]; γ-tocopherol [µmol/L].

**Table 4:** Comparative analysis in normo- (NG) and hyperglycemia (HG) groups of people with obesity (PWO). Data are expressed as mean ± SD. For within-group (p (X_1 vs. X_2)) and between-group (#p) comparisons of the differences between measurements from before the diet program (**X_1**) and after the diet program (**X_2**), a *t*-test was used.

Parameter	X	Normoglycemia NG (N = 18)	Hyperglycemia HG (N = 35)	#p
Weight loss		12.4 ± 10.2	9.2 ± 5.8	#0.24
VFA loss		21.1 ± 18.4	21.2 ± 21.6	#0.83
TBW loss		2.69 ± 3.1	2.26 ± 3.91	#0.81
SMM loss		2.11 ± 2.67	2.07 ± 3.70	#0.95
L-SOD	1	179.56 ± 16.41	179.28 ± 15.06	#0.95
	2	184.56 ± 21.00	173.13 ± 15.75	**#0.033**
p (X_1 vs. X_2)		0.41	0.11	
L-GPX	1	57.55 ± 10.08	64.03 ± 10.37	**#0.038**
	2	48.58 ± 14.87	54.51 ± 15.93	#0.20
p (X_1 vs. X_2)		**0.0034**	6.3*10^−7^	
L-GST	1	0.23 ± 0.09	0.21 ± 0.11	#0.56
	2	0.33 ± 0.11	0.23 ± 0.13	**#0.013**
p (X_1 vs. X_2)		**0.008**	0.33	
L-KAT	1	496.0 ± 77.7	460.9 ± 72.4	#0.12
	2	496.4 ± 78.57	477.4 ± 64.9	#0.36
p (X_1 vs. X_2)		0.82	0.11	
L-GR	1	8.79 ± 2.70	8.23 ± 1.76	#0.38
	2	9.40 ± 1.93	9.00 ± 1.84	#0.48
p (X_1 vs. X_2)		**0.064**	**0.0007**	
L-LPS	1	1232.1 ± 239.6	1037.1 ± 207.6	**#0.004**
	2	1031.2 ± 187.0	857.0 ± 236.0	**#0.010**
p (X_1 vs. X_2)		**0.024**	**0.00012**	
L-MDA	1	0.41 ± 0.10	0.35 ± 0.05	#0.21
	2	0.35 ± 0.05	0.32 ± 0.05	**#0.031**
p (X_1 vs. X_2)		**0.045**	**0.0004**	
Retinol	1	0.58 ± 0.16	0.64 ± 0.16	#0.20
	2	0.58 ± 0.17	0.63 ± 0.15	#0.34
p (X_1 vs. X_2)		0.84	0.59	
α-tocopherol	1	25.72 ± 7.93	26.51 ± 7.58	#0.74
	2	28.18 ± 10.09	26.27 ± 7.92	#0.45
p (X_1 vs. X_2)		**0.038**	0.81	
γ-tocopherol	1	1.53 ± 0.55	1.65 ± 0.62	#0.49
	2	1.74 ± 0.67	1.45 ± 0.51	#0.14
p (X_1 vs. X_2)		0.18	**0.015**	
α/γ-tocopherol ratio	1	18.26 ± 6.80	17.08 ± 5.02	#0.54
	2	18.48 ± 11.20	19.04 ± 5.25	#0.81
p (X_1 vs. X_2)		0.90	**0.008**	

Legend: NG—normoglycemia group; HG—hyperglycemia group; X_1—before WRP; X_2—after WRP; weight [kg]; VFA—visceral fat area [cm^2^]; TBW—total body water [L]; SMM—skeletal muscle mass [kg]; L-SOD—hemolysate superoxide dismutase [NU/mgHb]; L-GPX—hemolysate glutathione peroxidase [IU/gHb]; L-GST—hemolysate glutathione transferase [IU/gHb]; L-GR—hemolysate glutathione reductase [IU/gHb]; L-KAT—hemolysate catalase [kIU/gHb]; L-LPS—hemolysate lipofuscin [RF/gHb]; L-MDA—hemolysate malondialdehyde [µmol/gHb]; retinol [µmol/L]; α-tocopherol [µmol/L]; and γ- tocopherol [µmol/L].

## Data Availability

The database of aggregated statistics ready for analysis is stored in a secure, confidential, and password-protected repository on the server of the Medical University of Silesia. The data were anonymized. Completely non-identifiable records may be made available to interested persons/organizations upon request to jzalejskafiolka@sum.edu.pl.
